# Construction of an enzyme-constrained metabolic network model for *Myceliophthora thermophila* using machine learning-based *k*_*cat*_ data

**DOI:** 10.1186/s12934-024-02415-z

**Published:** 2024-05-15

**Authors:** Yutao Wang, Zhitao Mao, Jiacheng Dong, Peiji Zhang, Qiang Gao, Defei Liu, Chaoguang Tian, Hongwu Ma

**Affiliations:** 1 Key Laboratory of Industrial Fermentation Microbiology of the Ministry of Education, Tianjin Key Laboratory of Industrial Microbiology, College of Biotechnology, Tianjin University of Science and Technology, Tianjin, 300457 China; 2 Haihe Laboratory of Synthetic Biology, Tianjin, 300308 China; 3 Key Laboratory of Engineering Biology for Low-Carbon Manufacturing, Tianjin Institute of Industrial Biotechnology, Chinese Academy of Sciences, Tianjin, 300308 China; 4 Biodesign Center, Key Laboratory of Engineering Biology for Low-carbon Manufacturing, Tianjin Institute of Industrial Biotechnology, Chinese Academy of Sciences, Tianjin, 300308 China; 5 National Technology Innovation Center of Synthetic Biology, Tianjin, 300308 China

**Keywords:** Enzyme-constrained model, *Myceliophthora thermophila*, Metabolic engineering, Machine learning, Carbon source hierarchy utilization

## Abstract

**Background:**

Genome-scale metabolic models (GEMs) serve as effective tools for understanding cellular phenotypes and predicting engineering targets in the development of industrial strain. Enzyme-constrained genome-scale metabolic models (ecGEMs) have emerged as a valuable advancement, providing more accurate predictions and unveiling new engineering targets compared to models lacking enzyme constraints. In 2022, a stoichiometric GEM, iDL1450, was reconstructed for the industrially significant fungus *Myceliophthora thermophila*. To enhance the GEM’s performance, an ecGEM was developed for *M*. *thermophila* in this study.

**Results:**

Initially, the model iDL1450 underwent refinement and updates, resulting in a new version named iYW1475. These updates included adjustments to biomass components, correction of gene-protein-reaction (GPR) rules, and a consensus on metabolites. Subsequently, the first ecGEM for *M. thermophila* was constructed using machine learning-based *k*_*cat*_ data predicted by TurNuP within the ECMpy framework. During the construction, three versions of ecGEMs were developed based on three distinct *k*_*cat*_ collection methods, namely AutoPACMEN, DLKcat and TurNuP. After comparison, the ecGEM constructed using TurNuP-predicted *k*_*cat*_ values performed better in several aspects and was selected as the definitive version of ecGEM for *M. thermophila* (ecMTM). Comparing ecMTM to iYW1475, the solution space was reduced and the growth simulation results more closely resembled realistic cellular phenotypes. Metabolic adjustment simulated by ecMTM revealed a trade-off between biomass yield and enzyme usage efficiency at varying glucose uptake rates. Notably, hierarchical utilization of five carbon sources derived from plant biomass hydrolysis was accurately captured and explained by ecMTM. Furthermore, based on enzyme cost considerations, ecMTM successfully predicted reported targets for metabolic engineering modification and introduced some new potential targets for chemicals produced in *M. thermophila*.

**Conclusions:**

In this study, the incorporation of enzyme constraint to iYW1475 not only improved prediction accuracy but also broadened the model’s applicability. This research demonstrates the effectiveness of integrating of machine learning-based *k*_*cat*_ data in the construction of ecGEMs especially in situations where there is limited measured enzyme kinetic parameters for a specific organism.

**Supplementary Information:**

The online version contains supplementary material available at 10.1186/s12934-024-02415-z.

## Background

*Myceliophthora thermophila*, a thermophilic filamentous fungus, thrives at high temperatures (45–50 ℃) which possesses a remarkable ability to secrete various glycoside hydrolases and auxiliary oxidation enzymes, making it an efficient plant biomass degrader [1, 2]. These unique characteristics make *M*. *thermophila* a highly promising candidate for biotechnological applications in biomass conversion and high-temperature fermentation [2]. Notably, *M*. *thermophila* has been engineered to be a cell factory for the production of enzyme [3] and various chemicals, including fumarate [4], succinic acid, malate [5], malonic acid [6], 1,2,4-butanetriol [7] and ethanol [8, 9]. Additionally, efforts have been made to engineer this fungus to be an outstanding consolidated bioprocessing (CBP) strain to produce chemicals from biomass sources [5, 10].

Genome-scale Metabolic Models (GEMs) are effective tools for elucidating cellular phenotypes and predicting potential engineering targets to guide development of industrial strains [11–13]. In 2022, a manually curated genome-scale metabolic model, iDL1450, was constructed for *M*. *thermophila* [14]. With the help of iDL1450, an optimized rational design for important bulk chemicals was simulated and metabolic differences under different temperature conditions were analyzed. However, GEMs only consider stoichiometric constraints, which may not accurately capture the intracellular conditions. To enhance the performance of GEMs, the integration of enzyme constraints into the models has been explored. Several methods have been developed to incorporate the enzyme constraints, considering enzyme concentration, enzyme catalytic efficiency, and enzyme molecular weight [15–21]. In 2007, the first mathematical framework, Flux Balance Analysis with Molecular Crowding (FBAwMC), was established by Beg et al. taking into account enzyme constraints based on macromolecular crowding [22]. FBAwMC imposes constraints on enzyme concentrations at a physical level by introducing crowding coefficients, thereby achieving an overall constraint on enzyme activity. Subsequently, several methods, such as MOMENT [23], GECKO [24], AutoPACMEN [25], and ECMpy [17], has been developed to integrate enzyme constraints into GEMs.

In 2017, based on FBAwMC, Sánchez et al. developed the GECKO toolbox, which extends GEMs by adding new rows to the S-matrix that represent the enzymes and new columns representing each enzyme’s usage [24]. The enzyme-constrained GEM (ecGEM) ecYeast7 revealed improved predictive performance compared with the Yeast7 and identified enzyme limitation as a major driving force behind enzymatic protein reallocation. Bekiaris et al. Combined the MOMENT and GECKO methods to introduce AutoPACMEN, a method capable of automatically retrieving enzyme data from the BRENDA [26] and SABIO-RK [27] databases, marking a significant step in automating ecGEM construction [25]. Additionally, in 2021, Mao et al. presented ECMpy, an automated method for ecGEM construction, which simplified the workflow without modifying the S-matrix [17]. ECMpy was employed to construct an ecGEM for *Escherichia coli*, demonstrating improved prediction accuracy for various cellular phenotypes [17]. Subsequently, ECMpy was utilized to develop ecGEMs for *Bacillus subtilis* [20] and *Corynebacterium glutamicum* [19], providing more precise predictions and guiding the rational design of microbial cell factories.

In this study, we first updated and modified iDL1450 to a new version called iYW1475. Based on this GEM, an enzyme-constrained model for *M*. *thermophila* was constructed using the ECMpy workflow. During the construction, enzyme turnover numbers (*k*_*cat*_) were gathered in three distinct methods (AutoPACMEN [25], DLKcat [28] and TurNuP [29]), resulting in three versions of ecGEMs (eciYW1475_AP, eciYW1475_DL and eciYW1475_TN). After comparison, eciYW1475_TN was selected as the final ecGEM version for *M*. *thermophila* (ecMTM). Simulation of substrate hierarchy utilization and prediction of metabolic engineering targets were performed using ecMTM.

## Methods

### Measurements of RNA and DNA content in* M. thermophila*

In order to determine RNA and DNA content in *M. thermophila*, the wild-type strain ATCC 42464 was grown on Vogel’s minimal medium supplemented with 2% glucose (GMM) containing 1.5% agar at 35 ℃ for 7 days to obtain mature conidia. Conidia was inoculated into 50 mL GMM to a final concentration of 1×10^6^ conidia/mL in a 250-mL Erlenmeyer flask. Then, liquid cultures were incubated under 45 ℃ at 150 rpm in a rotary shaker for 20 h. RNA content was quantified using the method described in report [30, 31]. A 2 mL sample was collected and certificated at 10,000×g for 5 min. The resulting pellet was washed three times with 3 mL of cold 0.7 M HClO_4_. Subsequently, the mycelium was resuspended in 3 mL of 0.3 M KOH and incubated at 37 ℃ for 60 min with occasional shaking. Following cooling to room temperature, the samples were neutralized by adding 1.0 mL 3 M HClO_4_, followed by centrifugation. The supernatant was collected, and the pellet underwent two washes with 4 mL of cold 0.5 M HC1O_4_. The supernatants were combined, and the volume was adjusted to 15 mL with 0.5 M HClO_4_. Finally, the samples were clarified by centrifugation, and the absorbance A_*260nm*_ was measured using a UV spectrometry (Nanodrop 2000c spectrophotometer). For the determination of biomass dry weight, mycelium was collected using vacuum filtration, washed three times with distilled water, and rapidly frozen in liquid nitrogen. Then the biomass samples were lyophilized under − 40 ℃ until a constant weight was achieved.

To measure DNA content, approximately 0.01 g of lyophilized mycelium was ground into powder in liquid nitrogen in a mortar. Following this, 1 mL extraction buffer (200 mM Tris·HCI pH 8.5, 250 mM NaCl, 25 mM EDTA, 0.5% SDS) [32] was added and incubated at 60 ℃ for 30 min. The sample was subjected to two extractions by adding an equal volume of phenol:chloroform:isoamy lalcohol (25:24:1, v/v/v) with certification at 10,000×g for 10 min. The supernatant was mixed with 1/10 volume of 3 M sodium acetate (pH 5.3) and 2.5 volumes ethanol to precipitate DNA at − 20 ℃ for 1 h. After precipitation, the DNA was resuspended in TNE (1 M NaCl, 10 mM EDTA, 0.1 M Tris·HCl, pH 7.4) and treated with RNase, followed by a second precipitation and wash to remove degraded RNA. Finally, the DNA content was determined using a Nanodrop spectrophotometer.

## Model update and format modification

Prior to establishing ecGEM, the initial model iDL1450 underwent comprehensive updates and format modifications to align with ECMpy requirements. In detail, biomass components were adjusted based on experimental data (Additional file 1: Table S1). Precise corrections were applied to RNA, DNA, protein and amino acid content [33], according to measured data. Subsequently, the content of lipids and cell wall was adjusted according to literatures [34] and [30], respectively.

Some redundant metabolites were identified by their corresponding IDs and names and were manually consolidated into singular entities (Additional file 2: Table S2). Besides, gene-protein-reaction (GPR) relationships were checked and updated based on experiment data from previous reports [4, 7, 35] alongside KEGG annotation [36], mainly including glycolysis, tricarboxylic acid cycle (TCA) and oxidative phosphorylation pathways (Additional file 3: Table S3). After the revision, gene number was increased from 1450 to 1475 and the initial model was renamed iYW1475.

To meet requirement of ECMpy, several changes had been made. Primarily, metabolite names were mapped to those in BiGG database [37] using KEGG [36] identifier, CHEBI IDs [38] and metabolite names (Additional file 4: Table S4). Then the format of modified GEM was converted from XML to JavaScript Object Notation (JSON) format. Furthermore, protein IDs in UniProt database [39] were collected and integrated into JSON format model as a part of gene annotation.

## Construction of ecGEM and calibration of enzyme kinetic parameters

**Fig. 1:**
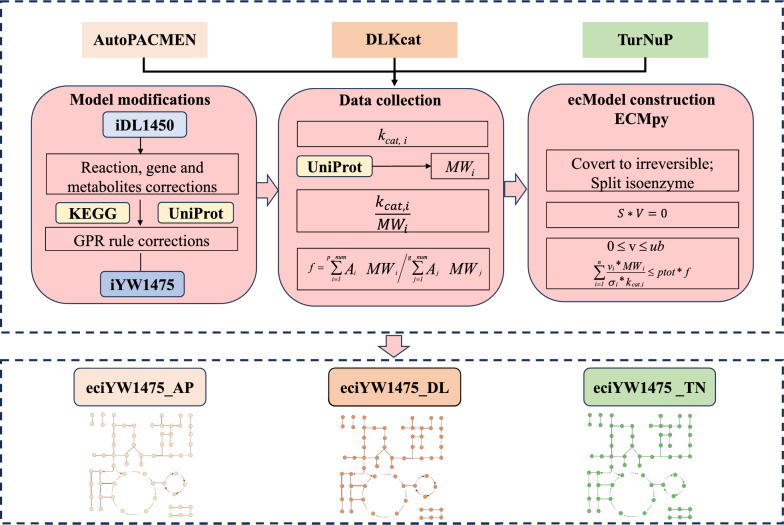
Workflow for the construction of eciYW1475_AP, eciYW1475_DL and eciYW1475_TN

The ecGEM was constructed based on iYW1475 using the ECMpy workflow (see Fig. 1). Initially, reversible reactions were transformed into pairs of irreversible reactions, and reactions catalyzed by multiple isoenzymes were segregated into distinct reactions. The enzyme mass fraction (*f*) was determined as 0.55, calculated using Eq. (1) based on unpublished proteomic data measured in our lab, where *A*_*i*_ and *A*_*j*_ represent the abundances (mole ratio) of the *i*-th protein (*p_num* represents proteins expressed in the model) and *j*-th protein (*g_num* represents proteins expressed in the whole proteome of *M. thermophila*), respectively; *MW*_*i*_ is molecular weight of an enzyme catalyzing reaction *i*. Additionally, enzyme subunit information was sourced from the ‘Interaction information’ section in the UniProt database (see Additional file 5: Table S5).

For enzyme kinetic parameter data, three methods were employed: AutoPACMEN was utilized to collect enzyme kinetic parameter data based on EC numbers, and machine learning-based tools (DLKcat [28] and TurNuP [29]) were used to predict enzyme kinetic parameter information. Finally, the enzyme-constrained model was developed by incorporating enzyme constraints (see Eq. 2) into GEM, where *v*_*i*_ represents flux of *i*-th reaction in the model; *σ*_*i*_ denotes the saturation coefficient for *i*-th enzyme, with an average value of 0.5 assigned to all enzymes; *ptot* represents the total protein fraction (0.4653) in *M*. *thermophila* and *f* represents the mass fraction of enzymes; *k*_*cat,i*_ represents the turnover number of the enzyme catalyzing reaction *i*.

1 $${\text{f}} = {{\sum\limits_{i = 1}^{p\_num} {\begin{array}{*{20}c} {\mathop A\nolimits_{i} } & {\mathop {MW}\nolimits_{i} } \\ \end{array} } } \mathord{\left/ {\vphantom {{\sum\limits_{i = 1}^{p\_num} {\begin{array}{*{20}c} {\mathop A\nolimits_{i} } & {\mathop {MW}\nolimits_{i} } \\ \end{array} } } {\sum\limits_{j = 1}^{g\_num} {\begin{array}{*{20}c} {\mathop {\text{A}}\nolimits_{j} } & {\mathop {MW}\nolimits_{j} } \\ \end{array} } }}} \right. \kern-0pt} {\sum\limits_{j = 1}^{g\_num} {\begin{array}{*{20}c} {\mathop {\text{A}}\nolimits_{j} } & {\mathop {MW}\nolimits_{j} } \\ \end{array} } }}$$

2 $$\sum\limits_{{i = 1}}^{n} {\frac{{\mathop v\nolimits_{i} * \mathop {MW}\nolimits_{i} }}{{\mathop \sigma \nolimits_{i} * \mathop k\nolimits_{{cat,i}} }}} \le ptot * f$$

To enhance the alignment between model predictions and experimental data, additional fine-tuning of the original *k*_*cat*_ values was necessary for the enzyme-constrained model. The iterative calibration for *k*_*cat*_ was carried out utilizing two methods outlined in ECMpy [17]: the enzyme usage method and the ^13^C flux consistency method, relying on measured ^13^C flux data [33].

## Phenotype phase plane (PhPP) analysis

Cells deploy distinct metabolic strategies contingent upon varying oxygen and glucose levels. To simulate this dynamic cellular responses, PhPP analysis was conducted to explore metabolic behavior [40]. During the analysis, uptake flux ranges for oxygen and glucose were set to 0–50 mmol/gDW/h and 0–10 mmol/gDW/h, respectively. Parsimonious Flux Balance Analysis (pFBA) was used for PhPP analysis with the objective of maximum biomass [41].

## Flux variability analysis (FVA)

Flux balance analysis (FBA) using linear programming with GEMs might generate diverse optimal solutions for the same objective value [42, 43]. To assess the changes in solution space after introducing enzyme constraint, flux variability ranges between iYW1475 and ecGEM were compared using FVA method [43] with some modifications [19]. Specifically, for reactions involving isozymes, the maximal flux variability range within the isozymes was used (Eq. (3)). For reversible reactions in ecGEMs, the corresponding flux variability ranges were solved using Eq. (4) [19], where $\mathop v\nolimits_{i,isoj}^{\max }$ and $\mathop v\nolimits_{i,isoj}^{\min }$ represent the maximum and minimum fluxes, respectively, of the *j*-th reaction among the reactions associated with isozyme *i*; $\mathop v\nolimits_{i}^{\max }$and $\mathop v\nolimits_{i}^{\min }$ represent the maximum and minimum fluxes of reaction *i*; $\mathop v\nolimits_{i,isoj}^{\max }$ and $\mathop v\nolimits_{i,REV}^{\min }$ represent the maximum and minimum fluxes of the reversible reaction *i*.

3 $$\mathop {FV}\nolimits_{{\text{i}}} = \max (\mathop v\nolimits_{i,isoj}^{\max } - \mathop v\nolimits_{i,isoj}^{\min } ),j \in m$$

4 $$\mathop {FV}\nolimits_{{\text{i}}} = (\mathop v\nolimits_{i}^{\max } - \mathop v\nolimits_{i}^{\min } ) - (\mathop v\nolimits_{i,REV}^{\max } - \mathop v\nolimits_{i,REV}^{\min } )$$

## Metabolic adjustment simulation

GEMs or ecGEMs were always employed to investigate and explicate metabolic adaptations [15–21]. In this study, metabolic adjustment was simulated using ecMTM solving by pFBA to maximize biomass production with substrate uptake rates ranging from 0 to 5 mmol/gDW/h. To further elucidate the metabolic adaptation to varying glucose uptake rates, detailed explorations were performed, encompassing alterations in biomass yield (Eq. 5), where $v_{biomass}$ represents the flux of biomass and $v_{glucose}$ denotes the glucose uptake rate, ${MW}_{glucose}$ is the molecular weight of glucose; enzyme efficiency for biomass synthesis ($\varepsilon _{{biomass}}$, Eq. 6), where ${\text{E}}_{{\text{min, biomass}}}$ was determined using the minimum enzyme amount algorithm of ECMpy for biomass synthesis; energy synthesis enzyme cost (Eq. 7), where $E_{reaction\,for\,ATP\,production,\,\,i} \,$ represents the enzyme level of the *i*-th ATP production reaction, and $V_{net\_generated\_ATP,\,i}$ represents the flux of the *i*-th ATP production reaction; and oxidative phosphorylation ratio (proportion of ATP produced by oxidative phosphorylation to the total ATP production) [20].

5 $${\text{Biomass\, yield}} = \frac{{\mathop v\nolimits_{{biomass}} }}{{\begin{array}{*{20}c} {\mathop v\nolimits_{{glu\cos e}} } & * & {\mathop {MW}\nolimits_{{glu\cos e}} } \\ \end{array} }}$$

6 $$\mathop {\varepsilon_{biomass} }\nolimits_{ \phantom{a} } = \frac{{\mathop {\mathop v\nolimits_{biomass} }\nolimits_{\phantom{a}} }}{{\mathop E\nolimits_{\min ,\,biomass} }}$$

7 $$Energy\,sysnthesis\,enzyme\,cost = \sum\nolimits_{i = 1}^{n} {\frac{{E_{reaction\,for\,ATP\,production,\,\,i} \,}}{{V_{net\_generated\_ATP,\,i} }}}$$

## Substrate hierarchy utilization measured in vivo

During cellular growth on various carbon sources, a hierarchy exists in their utilization. Utilization pattern of five carbon sources in *M*. *thermophila* was measured in this study. For measurement consumption of carbon sources, inoculation of *M*. *thermophila* ATCC42464 conidia into 100 mL 1 × Vogel’s minimal medium supplemented with five carbon sources (glucose, xylose, galactose, arabinose, cellobiose, 0.5% (w/v) for each substrate) to a final concentration of 1 × 10^6^ conidia/mL in a 250-mL Erlenmeyer flask. Then the liquid cultures were incubated at 45 ℃ and 150 rpm in a rotary shaker. Sample was centrifuged at 10,000×g for 5 min and the supernatant was filtered with 0.22-μm cellulose acetate syringe filters before analysis. The concentration of cellobiose was determined by HPLC (e2695; Waters, Manchester, United Kingdom) equipped with a Waters 2414 refractive index (RI) detector and an Aminex HPX-87H column (Bio-Rad) at 35 ℃; 5 mM H_2_SO_4_ was used as the mobile phase with constant flow rate 0.5 mL/min. Other carbon sources concentrations were determined by an ICS6000 high-performance anion exchange chromatography system equipped with a Dionex CarboPac™ PA200 column (3 × 250 mm), a pulsed amperometric detector (HPAEC-PAD) featuring a gold working electrode and a silver/silver chloride reference electrode (Thermo Fisher Scientific, Waltham, MA, USA). The column temperature was 30 ℃, the injection volume was 10 μL, and the flow rate was 0.3 mL/min. The mobile phases were 200 mM NaOH (A) and 10 mM NaOH (B).

## Substrate hierarchy utilization simulated in silico

In a recent study, Wang et al. developed a coarse-grained model to explain the sequential consumption of two carbon sources, providing a quantitative framework for microbial carbon source utilization [44]. Here, substrate hierarchy utilization of carbon source*s* for *M*. *thermophila* were explained using ecGEM in a quantitative framework. The enzyme efficiency in producing 12 biomass precursor metabolites G6P (Glucose 6-phosphate), F6P (Fructose 6-phosphate), G3P (Glyceraldehyde 3-phosphate), 3PG (Glycerate 3-phosphate), PEP (Phosphoenolpyruvate), PYR (Pyruvate), ACCOA (Acetyl-CoA), OAA (Oxaloacetate), AKG (2-Oxoglutarate), SUCC (Succinate), E4P (Erythrose 4-phosphate) and R5P (Ribose 5-phosphate) for five distinct carbon sources was calculated using ecMTM with substrate carbon atom uptake rate setting at 15 mmol/gDW/h. Then a comparative analysis was conducted to determine the order of substrate utilization. The enzyme efficiency for substrate synthesis precursors ($\mathop \varepsilon \nolimits_{biomass\,precursor}$) is calculated using Eq. (8):

8 $$\varepsilon_{{\text{biomass precursor}}} { = }\frac{{ \, v_{{\text{biomass precursor}}} }}{{{\text{E}}_{{\text{min, precursor}}} }}$$

where $\mathop v\nolimits_{biomass\,precursor}$ and $\mathop E\nolimits_{{\text{min, precursor}}}$ respectively represent the flux of biomass precursor and the minimum enzyme cost for biomass precursor synthesis.

## Prediction of metabolic engineering targets

A distinctive feature of ecGEM lies in its capability to calculate the enzyme cost of reactions, facilitating the identification of key enzymes within pathways [19]. In this study, potential modification targets were identified using two methods: one is enzyme cost-based sorting method (Method 1) in which the first 15 proteins were classified as top-demanded proteins and selected as potential targets for metabolic engineering; The second is the enzyme cost differences in different conditions (HGLP (high growth low product generation)/LGHP (low growth high product generation)) method (Method 2, reactions with a flux greater than 1 mmol/gDW/h are selected as potential targets for engineering modifications (HGLP involves the flux at biomass = 10%, while LGHP involves the flux at biomass = 100%) [19].

## Statistical analysis

All experiments were carried out in three independent repeated assays.

## Results

### Model update and format modification

The biomass components in iDL1450 were adjusted based on measured data for protein, RNA, and DNA content. Subsequent modifications of additional components were based on literature data, all of which are detailed in the supplementary file (Additional file 1: Table S1). After that, 96 duplicate metabolites were identified and consolidated into singular entities with identical identifiers and names (Additional file 2: Table S2). Additionally, gene-protein-reaction (GPR) rules, particularly reactions with ‘and’ relationships, were modified using information from literature and KEGG data. A total of 29 reactions with ‘and’ relationships in their GPR associations were revised (Additional file 3: Table S3). Through iterative corrections, a refined model, named iYW1475, was obtained containing 1475 genes, 2591 reactions, and 1700 unique metabolites. Additionally, format adjustments were executed to comply with ECMpy criteria, and leveraging multiple identifiers mapping, 55.2% of metabolite IDs were substituted with BiGG IDs (Additional file 4: Table S4). After these updates and format modifications, iYW1475 was transitioned from XML format to JSON format (all workflows are showed in Fig. 1).

## Construction of ecGEM for *M*.* thermophila*

**Fig. 2:**
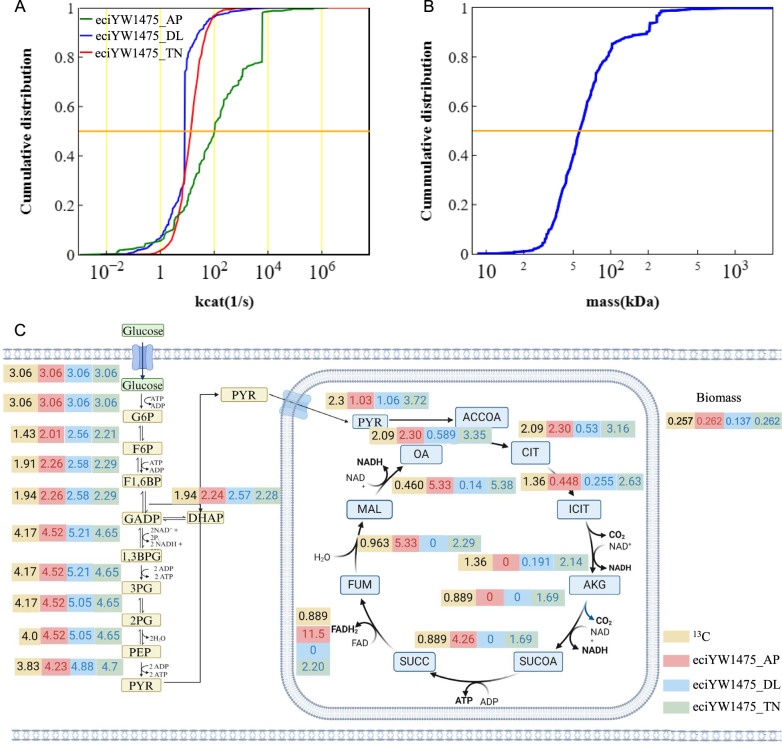
Basic information of eciYW1475_AP, eciYW1475_DL and eciYW1475_TN. **A** Cumulative distribution of *k*_*cat*_ values for three ecGEMs. **B** Cumulative distribution of molecular weights. **C** Flux comparison of three ecGEMs

Before integrating enzyme kinetic parameters, reactions in iYW1475 were transformed based on ECMpy workflow. After the transformation, the model was expanded to 6690 reactions (Table 1). Then, enzyme data were collected using AutoPACMEN method, accumulating a total of 4481 *k*_*cat*_ data ranging from 10^–3^ to 10^6^, leaving 1059 reactions without enzyme data. The missing values were filled with median value of all AutoPACMEN-collected *k*_*cat*_ data (Additional file 6: Table S6). Additionally, the molecular weights of the enzymes spanned from 8 to 1800 kDa (Fig. 2B). The constructed ecGEM from this workflow was denoted as eciYW1475_AP.

**Table 1:** Basic information of four models

Model	Reactions	Source of *k*_*cat*_	Number of *k*_*cat*_
iYW1475	2591	–	0
eciYW1475_AP	6690	BRENDA or SABIO-RK	4481
		Median imputation	1059
eciYW1475_DL	6690	DLKcat	3251
		BRENDA or SABIO-RK	52
		Median imputation	2237
eciYW1475_TN	6690	TurNuP	5519
		BRENDA or SABIO-RK	21

However, 19.1% of *k*_*cat*_ data cannot be collected through the AutoPACMEN method. Furthermore, only one *k*_*cat*_ value originated from the native species, while the remaining values were obtained from other species, indicating a low availability of data for enzyme kinetic parameters. Therefore, machine learning-based methods DLKcat and TurNuP were employed to predict *k*_*cat*_ data. DLKcat captured 67.7% *k*_*cat*_ data while TurNuP successfully predicted all *k*_*cat*_ values for reactions in iYW1475 (Additional file 6: Table S6). Consequently, a second ecGEM was constructed using DLKcat predicted data with missing values filled in using the median of DLKcat predicted *k*_*cat*_, resulting in eciYW1475_DL. Meanwhile, TurNuP-predicted data was chosen to construct a third ecGEM, named eciYW1475_TN. The distribution of *k*_*cat*_ values (Fig. 2A) indicated a more concentrated *k*_*cat*_ distribution in eciYW1475_TN and eciYW1475_DL, ranging from 10^−2^ to 10^3^ compared to those in eciYW1475_AP.

Next, *k*_*cat*_ values were calibrated using enzyme usage method until the growth rate approximated the measured value (Additional file 7: Table S7). After that, the ^13^C flux consistency method was employed to ensure flux consistency between ecGEM and measured fluxes (Additional file 7: Table S7). After the calibration of the *k*_*cat*_ values, growth and flux predictions were compared. Results showed that (Fig. 2C) the predicted biomass fluxes in eciYW1475_AP and eciYW1475_TN were both similar to experimental value. However, for eciYW1475_DL, the predicted biomass was much smaller than the experimental value due to missing values being filled with the median value of 7.8391 s^−1^ leading to an over-constrained model. Compared with eciYW1475_DL and eciYW1475_AP, eciYW1475_TN indicated a higher consistency with the ^13^C data (Fig. 2C, Additional file 8: Table S8). Discrepancies were observed between the predicted fluxes and the experimental data for eciYW1475_DL and eciYW1475_AP (Fig. 2C, Additional file 8: Table S8). It was noted that some predicted fluxes for the TCA pathway were zero when the glucose uptake rate was 3.0676 mmol/gDW/h. Considering *k*_*cat*_ coverage and performance of these three ecGEMs, the revised eciYW1475_TN was then used as the final version of ecGEM for *M. thermophila*, named ecMTM. All subsequent simulations were carried out with ecMTM.

## Introducing enzyme constraints reduced the solution space and improved prediction accuracy

**Fig. 3:**
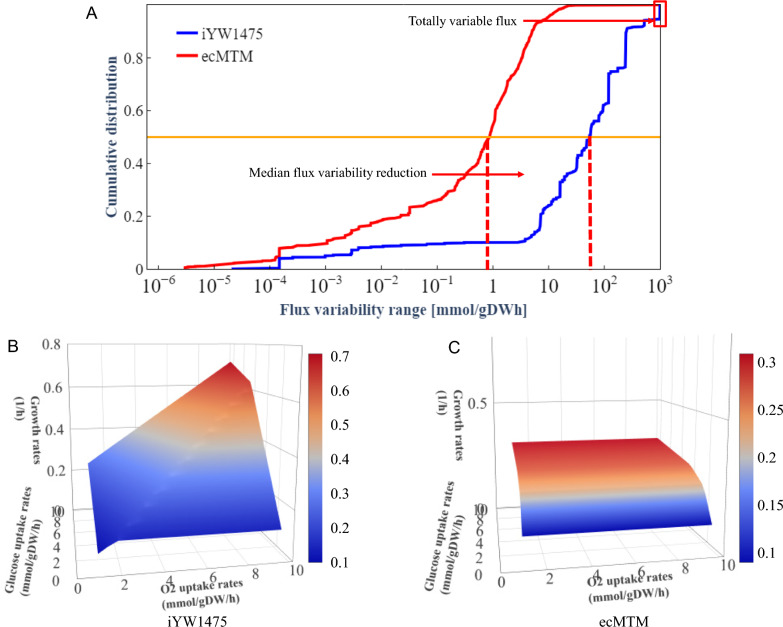
Simulation of solution space and growth rate. **A** Cumulative distribution of flux variability at high growth rates for iYW1475 and ecMTM. Simulation of growth rates at different glucose and oxygen uptake rates using iYW1475 (**B**) and ecMTM (**C**)

When a GEM is given a specific objective, solving it through FBA often results in alternate optimal solutions, which constitutes a limitation of GEMs [43]. To address this challenge and narrow the range of flux variability, various strategies, including the incorporation of enzyme constraints, have been explored in previous studies [17, 19–21]. In this study, the cumulative flux variability ranges between iYW1475 and ecMTM were compared using Flux Variability Analysis (FVA) with setting glucose uptake rate at 10 mmol/gDW/h. Results demonstrated that a substantial two-order reduction in the median flux variability range after introducing enzyme constraints. Furthermore, cumulative distribution indicated that there were 5% with totally variable flux (reactions that can carry any flux between − 1000 and 1000 mmol/gDW/h) in iYW1475, while no such extreme variability range was observed in ecMTM (Fig. 3A, Additional file 9: Table S9).

In experimental condition, growth rate of *M. thermophila* is 0.2573 h^−1^ with maximum glucose uptake rate of 3.0676 mmol/gDW/h when abundant oxygen and glucose are supplied [33]. The PhPP analysis of iYW1475 exhibited linear growth as a function of carbon source and oxygen uptake rates (Fig. 3B), which was inconsistent with experimental observations. In contrast, ecMTM constrained the maximal growth rate with increasing carbon source availability (Fig. 3C), indicating a significant reduction in the solution space. All these results suggest that incorporating enzyme constraints into GEMs make the simulation results closer to realistic cellular phenotypes.

## Simulating metabolic strategy adjustment

Enzyme-constrained models provide valuable insights into how cells adjust their metabolic pathways based on enzyme resources in response to increasing carbon source uptake. Previous studies have employed ecGEMs to simulate the metabolic adjustments in Yeast [24] and *E*. *coli* [17], elucidating overflow metabolism phenomena in these organisms. Simulation of metabolic strategy adjustment were conducted with a glucose uptake rate ranging from 0 to 6 mmol/gDW/h.

**Fig. 4:**
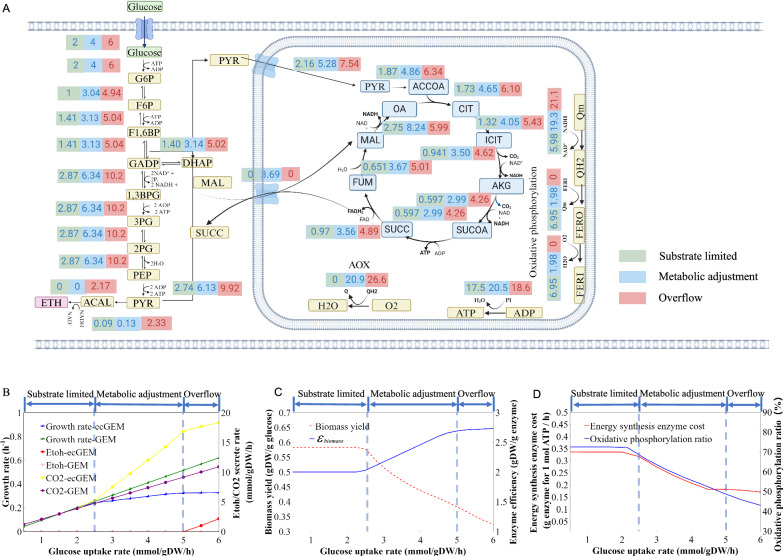
Simulation of metabolic adjustments. **A** Metabolic flux map of the three stages. **B** Comparison of metabolic adjustments between iYW1475 and ecMTM. **C** Trade-off phenomenon simulated by ecMTM. **D** Energy synthesis enzyme cost and energy production ratio of oxidative phosphorylation simulated by ecMTM

The results illustrated that metabolic pathways were dynamically adjusted with an increase in glucose uptake rate. These adjustments can be divided into substrate-limited, metabolic adjustment stage, and metabolic overflow stage (Fig. 4A). In the first stage (glucose uptake rate less than 2.5 mmol/gDW/h), the growth rate exhibited a linear relationship with glucose uptake, aligning with the behavior observed in iYW1475 (Fig. 4A, B). In the second stage (glucose uptake rate 2.5 to 5 mmol/gDW/h), due to the enzyme resource constraint, growth rate was decreased compared with iYW1475 along with the gradual increase of substrate supply (Fig. 4B). Meanwhile, metabolic pathways were adjusted to these with higher enzyme efficiency and more carbon loss resulting in lower biomass yield (Fig. 4B, C). Moving on to the third stage, as substrate supply continued to increase, a shift towards a more enzymatically efficient ethanol production pathway was observed. This shift resulted in an increase in ethanol production flux from 0 to 2.17 mmol/gDW/h. Overall, the results depicted a trade-off between enzyme efficiency and growth rate.

To investigate energy pathway regulation strategies during metabolic adjustments, energy synthesis enzyme costs and oxidative phosphorylation rates were calculated [20]. Cells showed a preference for energy-efficient respiratory oxidative phosphorylation at low growth rates, particularly in the substrate limited stage (Fig. 4D, Additional file 10: Table S10). However, as the glucose uptake rate increased during the metabolic adjustment stage, the ratio of oxidative phosphorylation with high enzyme consumption decreased, resulting in a decline of enzyme cost for energy synthesis (Fig. 4D, Additional file 10: Table S10). Due to enzyme limitation, more energy was produced through the glycolysis pathway. The regulation of the energy pathway indicated that enzyme limitation is a major driving force for the reallocation of enzymatic proteins for energy production.

## Simulation of substrate hierarchy utilization

As a highly efficient cellulose and hemicellulose degrader,* M*. *thermophila* exhibits proficiency in utilizing various oligosaccharides and monosaccharides derived from plant biomass hydrolysis. The primary degradation products include cellobiose, glucose, xylose, arabinose and galactose [45]. Investigating the preferential utilization of five substrates in *M. thermophila*, essential for environmental adaptation, was performed by simulating hierarchical substrate utilization using ecMTM from the perspective of enzyme efficiency (substrate carbon atom uptake was scaled to 15 mmol/gDW/h). According to the assessment principles [44], if the enzyme efficiency for synthesizing biomass precursors from one substrate is higher than that from another substrate, the former is sequentially consumed before the latter; otherwise, they are deemed to be simultaneously consumed (co-utilization).

**Fig. 5:**
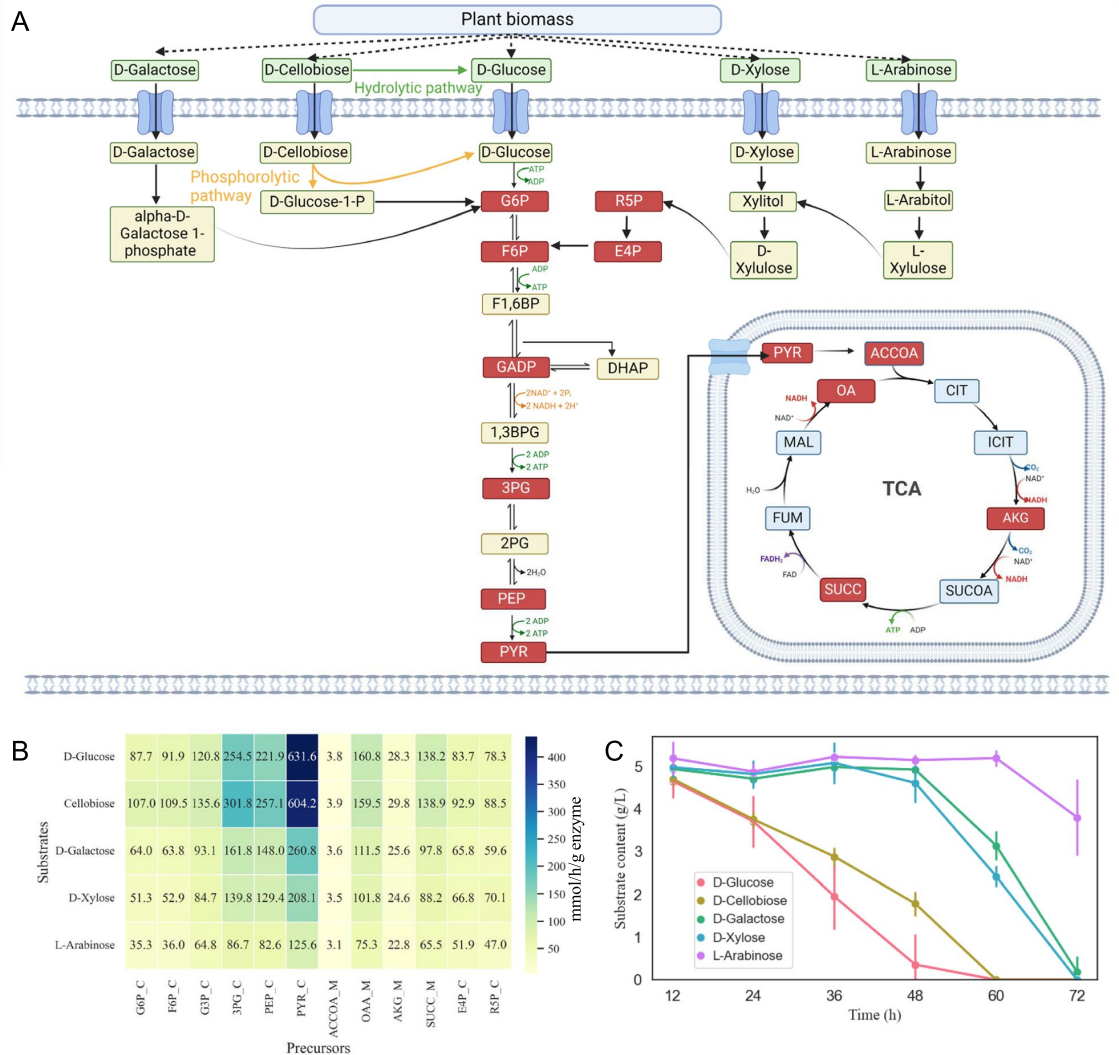
Substrate hierarchy utilization simulation. **A** Substrate utilization pathways for five carbon resources. Metabolites with red background represent 12 biomass precursors. **B** Simulation of substrate hierarchy utilization. Numbers represent enzyme efficiency. **C** Measured data of substrate utilization

There are mainly two pathways for cleaving cellobiose in *M*. *thermophila*, namely, hydrolytic pathway and phosphorolytic pathway [35] (Fig. 5A). In the hydrolytic pathway, β-glucosidase catalyzes one cellobiose molecule into two molecules glucose, while in the phosphorolytic pathway, cellobiose phosphorylase converts one cellobiose molecule into one glucose molecule and one glucose-1-phosphate (G1P) molecule. Glucose generated in these pathways is further catalyzed into G6P with consuming ATP whereas G1P, produced in the phosphorolytic pathway, is catalyzed into G6P by phosphoglucomutase without ATP consumption. In comparison to the hydrolytic pathway, 11 biomass precursors, excluding PYR, are synthesized from one cellobiose molecule through the phosphorolytic pathway, which consumes one less molecule of ATP. This reduction in ATP usage saves enzyme resources by 12.5% (in the case of G6P), as ATP production involves large amounts of enzyme resource through oxidative phosphorylation (Additional file 11: Table S11). As a result, there is a higher enzyme efficiency in synthesizing 11 biomass precursors from cellobiose compared with glucose (Fig. 5B). However, the hydrolytic pathway is chosen for pyruvate synthesis from cellobiose since ATP production and consumption are balanced through this pathway. The cellobiose hydrolytic pathway requires an additional β-glucosidase, accounting for 4.5% of the total enzyme resources, leading to lower enzyme efficiency in PYR synthesis compared with glucose. Meanwhile, enzyme efficiencies for synthesizing all the 12 biomass precursors with cellobiose and glucose are higher than those with xylose, galactose and arabinose. Therefore, glucose and cellobiose are first co-utilized showing a preferential utilization for the other three sugars.

Concerning galactose and xylose, the synthesis enzyme efficiency with the former is higher than that of the latter for 10 biomass precursors and is lower for 2, indicating a co-utilization relationship between the two sugars (Fig. 5B). Additionally, all enzyme efficiencies for the 12 precursors with xylose and galactose are higher than that of arabinose, resulting in arabinose being utilized last. Compared with xylose, there are two additional enzyme reactions for biomass precursor synthesis using arabinose which constitute 4.7% (in the case of G6P) of the total enzyme resources, resulting in lower enzyme efficiency (Additional file 11: Table S11). Overall, these simulation results are consistent with experimental measurements illustrated in Fig. 5C. The quantitative simulation elucidates the hierarchical utilization of different carbon sources derived from plant biomass in this fungus from the perspective of protein resource allocation.

## Exploration of metabolic engineering targets based on enzyme cost

**Fig. 6:**
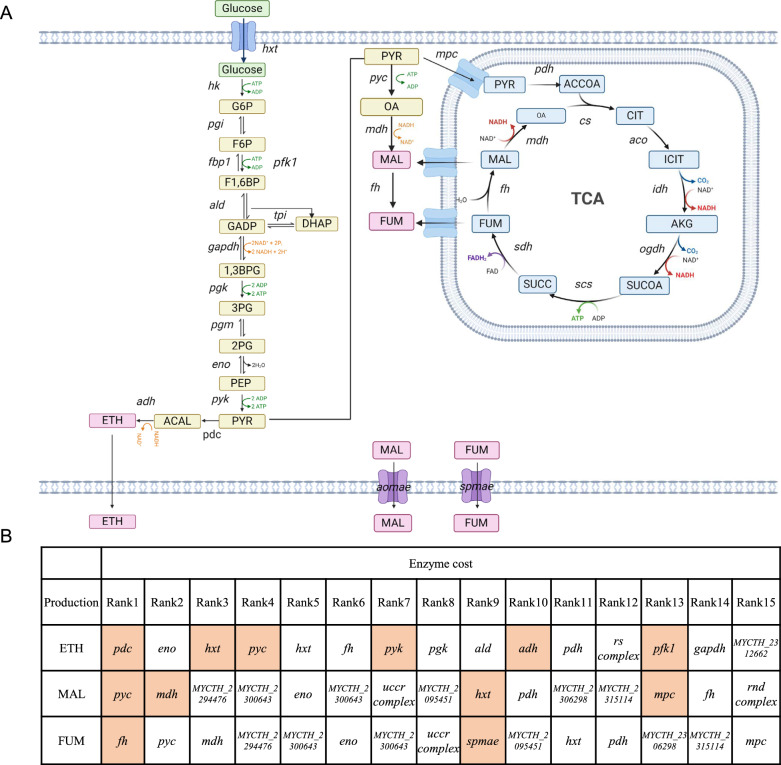
Metabolic engineering targets prediction for ethanol, malate and fumarate in* M*. *thermophila*. **A** Depicts the biosynthetic pathways for ethanol, malate and fumarate in *M*. *thermophila*. **B** Metabolic engineering targets for three products predicted using ecMTM. The targets reported in the literature are highlighted in orange. The reactions *uccr* (Ubiquinol-cytochrome c reductase complex), *rs* (RNA synthesis complex), and *rnd* (Respiratory-chain NADH dehydrogenase complex) are involved multiple genes

GEMs function as valuable predictive tools for identifying potential targets in metabolic engineering. In the case of *B. subtilis*, an ecGEM was applied to identify novel gene deletion targets, resulting in an increased yield of poly-γ-glutamic acid [18]. Based on enzyme costs, two methods (outlined in Method section) were employed to predict potential targets for three products: ethanol, malate and fumarate. The biosynthetic pathways for these three products were illustrated in Fig. 6A. In total, 12 out of 32 previously overexpressing targets were predicted by ecMTM using the first method as depicted in Fig. 6B. Modifying these 12 targets showed notable improvements in the production of target chemicals [4, 5, 8, 9].

Target prediction method 2 presents a modification strategy that includes both enhancing and weakening targets (Additional file 12: Table S12). Six enhancing targets for ethanol were successfully predicted and verified by overexpression target genes [8, 9]. Among these predicted targets, both *pdc* (pyruvate decarboxylase encoding gene) and *adh* (alcohol dehydrogenase encoding gene) were identified by these two methods, representing the final crucial steps in ethanol synthesis. Overexpressing PDC, which decarboxylates pyruvate to acetaldehyde, and ADH, which reduces acetaldehyde to ethanol, can directly enhance the ethanol synthesis capability. This strategy has been supported by previous studies [8, 9]. Additionally, two new enhancing targets, *eno* and *gapdh*, were predicted but have not been modified yet.

For malate, four enhancing targets were identified, including *pyc* (pyruvate carboxylase encoding gene), *mdh* (malate dehydrogenase encoding gene), and *hxt* (hexose transporter encoding gene), predicted by both methods. In microbes, PYC catalyzes the conversion of pyruvic acid to oxaloacetic acid with the fixation of CO_2_; the oxaloacetic acid is then converted into malate by MDH. PYC and MDH are two key steps in produce of malate through the reductive tricarboxylic acid (rTCA) pathway in cytoplasm [5]. Due to limited experiments on fumarate, only two predicted targets by these two methods were verified, leaving the remaining targets serve as potential objectives for fumarate modification.

Notably, among the predicted targets, *hxt* and *eno* were shared by these three chemicals. HXT is responsible for transporting glucose, a key factor in increasing glucose uptake. This has been confirmed by studies showing that overexpressing HXT can elevate ethanol and malate yield [5, 8, 9]. As a vital step of glycolysis, ENO is regarded as a key enzyme due to its low *k*_*cat*_. Therefore, *eno* is a potential target to increase precursor metabolite flux from glycolysis for enhancing product synthesis, which is worth trying in future.

In the results predicted by method 2, putative oxaloacetate transporter was identified as a weakening target which contributes to the enhancement of malate and fumarate production. Knocking out this target in *Aspergillus carbonarius* has been shown to boost malate production [46], making it a prospective modification target for improving malate synthesis. In addition, most of predicted weakening targets for these three products were focused on the respiratory chain, suggesting that modifying these reactions may allocate more enzyme resources for product synthesis.

## Discussion

In this research, an enzyme-constrained model ecMTM was constructed based on iDL1450 under the ECMpy framework. Enzyme turnover number *k*_*cat*_ is a fundamental parameter in the quantitative study of enzyme activity and is crucial for understanding cellular metabolism, physiology, and resource allocation [15–21]. Several databases such as BRENDA, UniProt, and SABIO-RK offer enzyme parameter queries, while automated pipelines like AutoPACMEN facilitate the automated acquisition of *k*_*cat*_ data for ecGEMs. Besides, *k*_*cat*_ values can be predicted through methods based on machine learning algorithms, such as DLKcat and TurNuP. During construction of ecMTM, three versions of ecGEMs were developed using *k*_*cat*_ values obtained through AutoPACMEN, DLKcat and TurNuP. After comparison, ecGEM constructed using TurNuP-predicted data performed better in several aspects and was selected as the final version of ecGEM for *M*. *thermophila*. Nowadays, the machine learning based methods has gradually used in ecGEMs with improved performance [28, 47].

The constructed ecMTM demonstrated improved accuracy in simulating strain behavior compared to iYW1475. Incorporating enzyme constraints into iYW1475 notably reduced the solution space, yielding predictions closer to experimental observations. However, there are still some disagreements that could not explain by ecMTM. In particular, metabolic strategy adjustment simulations within the model revealed an ethanol overflow, as was observed in yeast ecGEM [24]. Although ethanol overflow is important for biofuel production from plant biomass, it is not observed in *M*. *thermophila* wild type strain. These discrepancies suggest that factors beyond enzyme constraints influence cell phenotype. Nonetheless, metabolic adjustment simulation provided insight into potential strategies to increase ethanol flux.

Due to enzyme resource limitations and quantified enzyme efficiencies, ecMTM could predict hierarchy substrate utilization in a quantitative perspective. *M*. *thermophila* typically found in low-carbon-content soil environments requires lignocellulolytic enzymes for lignocellulose degradation [48]. In this niche, the utilization of carbon sources with low cost of enzyme is important for the survival of *M*. *thermophila*. Hierarchy substrate utilization was determined in liquid shake flask and simulated with ecMTM. The simulation unveiled the enzyme-cost-driven hierarchy in substrate utilization by *M*. *thermophila*. In previously research, Ramkrishna et al. [49] developed a cybernetic model to simulate mixed-substrate growth dynamics of *E. coli*. This model utilizes dynamic equations to describe the rates of individual reactions in metabolic pathways, taking into account substrate concentration, enzyme concentration and enzyme activity. It requires a large amount of experimental data to fit model parameters. On the other hand, Enzyme efficiency method determines the hierarchical utilization of carbon sources by calculating the enzyme efficiency for biomass precursors without relying on complex dynamic equations or experimental data. Nevertheless, it ignores the dynamic regulation processes in metabolic pathways. Therefore, the cybernetic model is better suitable for studying the dynamic regulation processes of metabolic pathways in biological systems, while the enzyme efficiency method is more suitable for predicting substrate hierarchy utilization.

Based on enzyme costs, metabolic engineering targets were predicted using ecMTM. Notably, the first prediction method revealed several shared targets across the three products, including *hxt* and *eno*. Similarly, the second prediction method predominantly highlighted targets within the glycolysis pathway to enhance these products. These predictions underscore the pivotal role of glucose transport and the rapid glycolysis flux in driving the production of these specific products.

After integration of enzyme constraints, the predictive capabilities of GEM were significantly enhanced. As we all know, biological systems are extremely complex with various constraints and hierarchical regulations. Enzyme constraints, though influential, might not comprehensively capture the intricacies of these systems. Furthermore, *M*. *thermophila* exhibits special phenotypes, such as thermophily and excellent ability of cellulose degradation. To elucidate these characteristics, it may be necessary to introduce additional constraints, such as temperature constraints, or development of comprehensive metabolic network models like ME models [50] and etcGEM models [51].

## Conclusion

In this study, an enzyme-constrained genome-scale metabolic model ecMTM was constructed for *M*. *thermophila* within ECMpy framework using machine learning-based *k*_*cat*_ data predicted by TurNuP. Simulation results demonstrated that enzyme constraints could reduce the model’s solution space, leading to more realistic pathway predictions. The ecMTM successfully predicted and elucidated the hierarchical utilization of five carbon sources derived from lignocellulose. Moreover, key enzymes were identified for three chemicals, providing rational metabolic engineering modifications to increase the yield of valuable compounds.

## Supplementary Information

Additional file 1: Table S1. Biomass composition.

Additional file 2: Table S2. Metabolite consensus.

Additional file 3: Table S3. Refined GPR associations.

Additional file 4: Table S4. Metabolite ID mapping to BiGG ID.

Additional file 5: Table S5. Subunit number information form UniProt.

Additional file 6: Table S6. *k*_*cat*_ data obtained from three methods.

Additional file 7: Table S7. Calibration of *k*_*cat*_ for three ecGEMs by two methods.

Additional file 8: Table S8. Flux comparison of three ecGEMs.

Additional file 9 Table S9. FVA of iYW1475 and ecMTM.

Additional file 10: Table S10. ATP generation in three stages.

Additional file 11 Table S11. Reaction fluxes and enzyme amounts for precursors synthesis of 12 biomass precursors from five carbon sources.

Additional file 12 Table S12. Targets prediction by enzyme allocation method.

## Data Availability

The models and code used in this study can be found at: https://github.com/wangtao-cell/ecMTM.

## Abbreviations

GEMs: Genome-scale metabolic models
ecGEMs: Enzyme-constrained genome-scale metabolic models
GPR: Gene-protein-reaction
CBP: Consolidated bioprocessing
GMM: Vogel’s minimal medium supplemented with 2% glucose
TCA: Tricarboxylic acid cycle
rTCA: Reductive tricarboxylic acid cycle
PhPP: Phenotype phase plane
pFBA: Flux balance analysis
FBA: Flux balance analysis
FVA: Flux variability analysis
G6P: Glucose 6-phosphate
F6P: Fructose 6-phosphate
G3P: Glyceraldehyde 3-phosphate
3PG: Glycerate 3-phosphate
PEP: Phosphoenolpyruvate
PYR: Pyruvate
ACCOA: Acetyl-CoA
OAA: Oxaloacetate
AKG: 2-Oxoglutarate
SUCC: Succinate
E4P: Erythrose 4-phosphate
R5P: Ribose 5-phosphate
G1P: Glucose-1-phosphate
DW: Dry cell weight
